# Screening, simulation, and optimization design of small molecule inhibitors of the SARS-CoV-2 spike glycoprotein

**DOI:** 10.1371/journal.pone.0245975

**Published:** 2021-01-25

**Authors:** Chuancai Sun, Jian Zhang, Jiao Wei, Xiaoli Zheng, Xianyang Zhao, Zengjun Fang, Dongmei Xu, Huiqing Yuan, Yipeng Liu

**Affiliations:** 1 Department of Nephrology, The First Affiliated Hospital of Shandong First Medical University & Shandong Provincial Qianfoshan Hospital, Jinan, China; 2 Department of Nephrology, Shandong Provincial Qianfoshan Hospital, Shandong University, Jinan, China; 3 Institute of Medical Sciences, The Second Hospital, Cheeloo College of Medicine, Shandong University, Jinan, China; 4 Department of Neurosurgery, The First Affiliated Hospital of Shandong First Medical University & Shandong Provincial Qianfoshan Hospital, Jinan, China; 5 Department of Emergency, Shandong Provincial Hospital Affiliated to Shandong First Medical University, Jinan, China; 6 Department of Pharmacy, The Second Hospital, Cheeloo College of Medicine, Shandong University, Jinan, China; 7 Nephrology Research Institute of Shandong Province, Jinan, China; University of Akron, UNITED STATES

## Abstract

The severe acute respiratory syndrome coronavirus 2 (SARS-CoV-2) outbreak is a public health emergency of international concern. The spike glycoprotein (S protein) of SARS-CoV-2 is a key target of antiviral drugs. Focusing on the existing S protein structure, molecular docking was used in this study to calculate the binding energy and interaction sites between 14 antiviral molecules with different structures and the SARS-CoV-2 S protein, and the potential drug candidates targeting the SARS-CoV-2 S protein were analyzed. Tizoxanide, dolutegravir, bictegravir, and arbidol were found to have high binding energies, and they effectively bind key sites of the S1 and S2 subunits, inhibiting the virus by causing conformational changes in S1 and S2 during the fusion of the S protein with host cells. Based on the interactions among the drug molecules, the S protein and the amino acid environment around the binding sites, rational structure-based optimization was performed using the molecular connection method and bioisosterism strategy to obtain Ti-2, BD-2, and Ar-3, which have much stronger binding ability to the S protein than the original molecules. This study provides valuable clues for identifying S protein inhibitor binding sites and the mechanism of the anti-SARS-CoV-2 effect as well as useful inspiration and help for the discovery and optimization of small molecule S protein inhibitors.

## 1. Introduction

In December 2019, patients with unexplained pneumonia were reported in Wuhan, Hubei Province, China; later, the pneumonia was confirmed to be caused by severe acute respiratory syndrome coronavirus 2 (SARS-CoV-2), and the induced disease was termed coronavirus disease 2019 (COVID-19) [1–3]. Within a few months, SARS-CoV-2 quickly spread around the world. On January 30, 2020, the World Health Organization (WHO) declared that the SARS-CoV-2 outbreak constituted a public health emergency of international concern (PHEIC). Over 3.6 million new COVID-19 cases and 69000 new deaths were reported to the WHO for the week ending 20 December. A cumulative total of over 40 million cases and 1.1 million deaths have been reported thus far [4]. SARS-CoV-2 has a long incubation period and strong infectiousness, and the general population is susceptible, which brings unprecedented challenges to global public health. Its prevention and treatment are of vital importance, but unfortunately, there are still no effective drugs; therefore, the screening and optimization design of drugs targeting SARS-CoV-2 are particularly critical.

To date, a number of drugs have been reported to have an anti-SARS-CoV-2 effect, including remdesivir (prodrug of remdesivir triphosphate, EC_50_ = 0.77 μM, CC_50_ > 100 μM, SI > 129.87), which has been previously used to treat Ebola virus. In the United States, it has completed a phase III clinical trial as a drug for treating SARS-CoV-2 [5–7]. Oseltamivir (prodrug of oseltamivir carboxylate), commonly used to treat influenza viruses, has also been used in studies against SARS-CoV-2 [8]. Zhu et al. found that arbidol (with an antiviral effect of 100% after 14 days of treatment) and lopinavir/ritonavir (with an antiviral effect of 55.9% after 14 days of treatment) also had a significant anti-SARS-CoV-2 effect [9]. Darunavir and hydroxychloroquine were proven to have a significant anti-SARS-CoV-2 effect [10]. According to Wang et al., favipiravir (prodrug of favipiravir-4-ribofuranosyl-5’-monophosphate and favipiravir-4-ribofuranosyl-5’-triphosphate, EC_50_ = 61.88 μM, CC_50_ > 400 μM, SI > 6.46) [11], ribavirin (prodrug of ribavirin 5’-monophosphate, EC_50_ = 109.50 μM, CC_50_ > 400 μM, SI > 3.65) [12, 13], nitazoxanide (prodrug of tizoxanide, EC_50_ = 2.12 μM, CC_50_ > 35.53 μM, SI > 16.76) [14, 15], and chloroquine (EC_50_ = 1.13 μM, CC_50_ > 100 μM, SI > 88.50) [5, 16] also had certain anti-SARS-CoV-2 effects. In addition, in a simulation study, some researchers investigated the binding between various functional proteins of SARS-CoV-2 with saquinavir, remdesivir, dolutegravir, and bictegravir [17]. Although initial progress has been made in drugs against SARS-CoV-2, most of the results have been obtained from in vitro or computational studies under specific conditions, and there is still much work to be done to improve the drugs before they can be used clinically. Whether the currently reported drugs can bind to the functional proteins of SARS-CoV-2, how effective the binding effect is, and the location of the binding sites have not yet been reported and are crucial for research on drugs against SARS-CoV-2.

**Fig 1:**
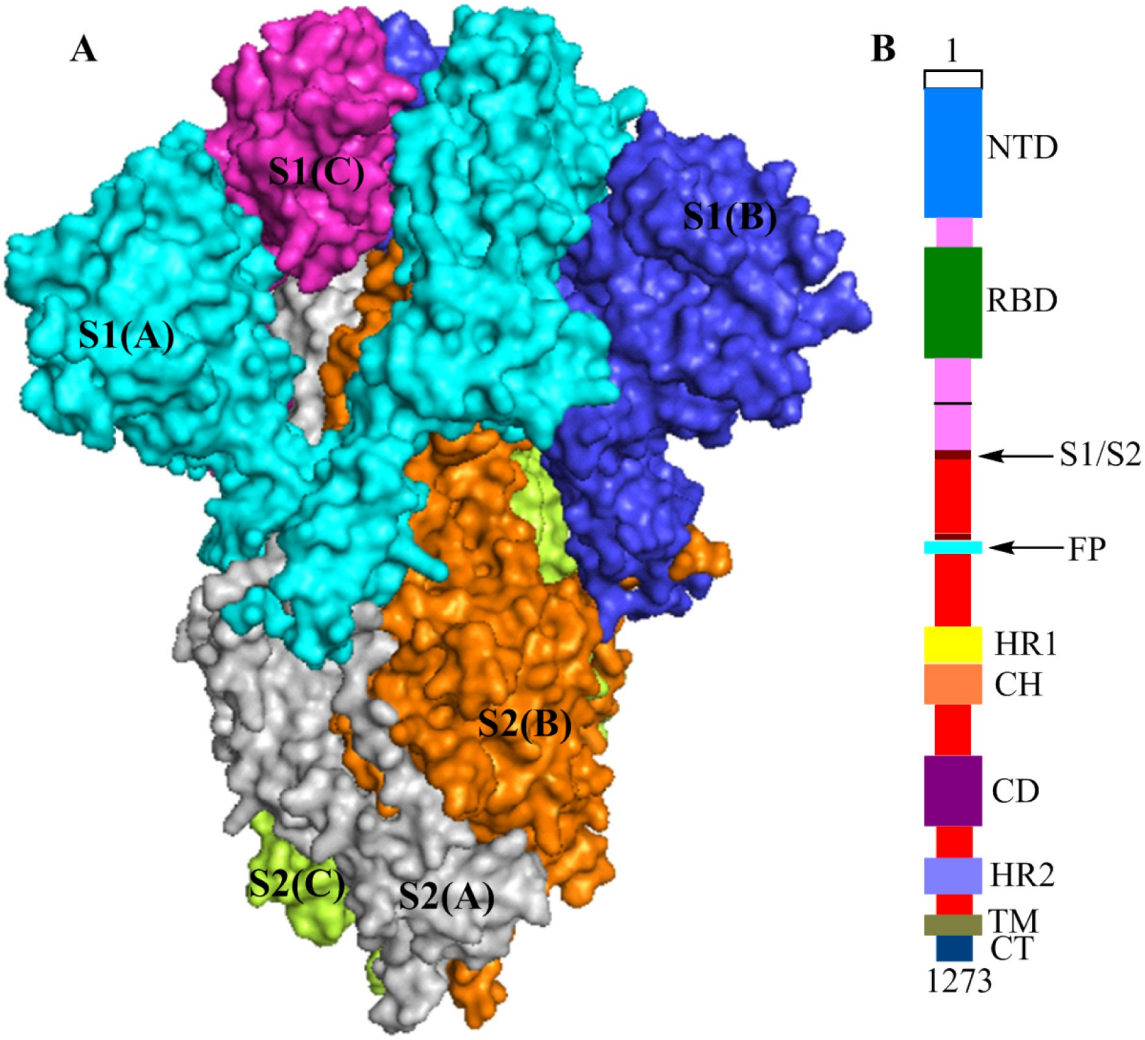
**(A)** The structures of the S protein trimer (electrostatic potential surface area (PDB ID: 6vxx)),S1(A), S2(A), S1(B), S2(B), S1(C) and S2(C) are shown in cyan, gray, blue, orange, magenta, and yellow-green, respectively. **(B)** Schematic of the SARS-CoV-2 S protein primary structure, colored by domain.

SARS-CoV-2 is a positive-strand RNA virus [18], and the spike glycoprotein (S protein) is an important homotrimeric surface antigen glycoprotein on SARS-CoV-2 [19]. Each monomer contains more than 1,200 amino acid residues and is divided into the S1 and S2 subunits (Fig 1A). Based on biological function, S1 contains the N-terminal domain (NTD, Val121-Thr305) and the receptor-binding domain (RBD, Pro330-Pro521). The sequence structure of S2 contains fusion peptide (FP, Thr816-Phe833), heptad repeat 1 (HR1, Gly908-Asp985), central helix (CH, Lys986-Gly1035), connector domain (CD, Thr1076-Leu1141), heptad repeat 2 (HR2, Asp1163-Lys1211), transmembrane domain (TM, Trp1212-Leu1234), and cytoplasmic tail (CT, Cys1235-Thr1273) (Fig 1B) [20, 21]. Similar to severe acute respiratory syndrome coronavirus (SARS-CoV), the most important function of the SARS-CoV-2 S protein is to attach to host cells and mediate fusion between the viral membrane and host cell membrane [22–24]. The process occurs as follows. The SARS-CoV-2 S protein binds to human angiotensin-converting enzyme 2 (ACE2) via the RBD, and the S protein undergoes a conformational change, causing the S protein trimer in the prefusion conformation to become unstable. The trimer sheds the S1 subunit, and the S2 subunit changes to a more stable postfusion conformation from the prefusion conformation to achieve fusion between the viral membrane and the host cell membrane, allowing the virus to enter into the host cell [25]. In this process, the RBD of S1 and the FP, HR1, CH, CD, HR2, TM and CT of S2 all play important roles. Notably, similar to SARS-CoV, the SARS-CoV-2 S protein also has obvious unique features in terms of biological functions. The affinity between ACE2 and the RBD of SARS-CoV-2 S1 is approximately 10- to 20-fold higher affinity than that of the RBD of SARS-CoV, which may be one of the reasons for the significant increase in SARS-CoV-2 infectiousness and infectivity [26]. Additionally, the HR1 sequence in SARS-CoV-2 S2 has high helix stability and can interact with the HR2 sequence to form a six-helix bundle structure, which makes the fusion ability of SARS-CoV-2 to host cells stronger than that of SARS-CoV [26].

In summary, the SARS-CoV-2 S protein plays a crucial role in the processes of viral adhesion, fusion and entry into host cells and is an ideal target for the development of anti-SARS-CoV-2 drugs. Based on the SARS-CoV-2 S protein, this study first screened candidate drugs with excellent binding ability to the S protein by comprehensively using different techniques, such as simulation, structural chemistry, drug design and targeted drug research, and then performed reasonable structural optimization on the screened drugs. The goal of this research is to provide data support for other researchers and contribute to the early discovery of specific anti-SARS-CoV-2 drugs.

## 2. Methods

To explore the binding site and the binding effect of antiviral drugs (dolutegravir, bictegravir, darunavir, saquinavir, lopinavir, arbidol, tizoxanide, chloroquine, hydroxychloroquine, oseltamivir carboxylate, remdesivir triphosphate, ribavirin 5’-monophosphate, favipiravir-4-ribofuranosyl-5’-monophosphate, and favipiravir-4-ribofuranosyl-5’-triphosphate) with the SARS-CoV-2 S protein, AutoDock4.2 [27] molecular modeling simulation software for protein-ligand docking was used. The structures of the S protein were obtained from the Protein Data Bank [28] (PDB ID: 6vxx [29]). We conducted docking studies with the flexible ligand (the above drugs) and the rigid receptor (S protein). The three-dimensional coordinates of the drug molecules were downloaded from the Drug Bank database.

A Lamarckian genetic algorithm (LGA) [30, 31] was used to generate binding poses. The docking was initiated with 150 randomly positioned poses, a maximum of 2.7×10^7^ generations, and a maximum number of 2.5 × 10^7^ energy evaluations. The rate of gene mutation, rate of crossover, and elitism parameters were set to 0.02, 0.80, and 1, respectively. For each S protein-drug docking, 30 docked poses were generated. The best binding energy conformation was selected for further analysis. Default binding site analyses of potential protease inhibitors of SARS-CoV-2 using AutoDock4.2 settings were used for all other parameters. The PyMol2.3 package was used to visualize the binding interactions between these ligands and a 3D model of a protease of SARS-CoV-2.

For the molecular simulation results, the three drug molecules with high binding energy and appropriate binding sites were selected for a systematic analysis of drug-S protein interactions. According to the protein structure and the distribution characteristics and polarity characteristics of amino acid residues around the binding sites of drug molecules, three selected drug molecules were subjected to rational structure-based optimization by the molecular connection method and bioisosterism strategy. The new molecules obtained after optimization were simulated and analyzed by the same method to obtain molecules with higher binding energies and closer interactions.

## 3. Results and discussion

### 3.1. Molecular docking results and discussion

#### 3.1.1. Molecular docking results

Table 1 shows the highest binding energy of each selected drug molecule with the S protein by molecular docking simulation. In the simulation of small molecules and the S protein, which was based on the biological function of S protein functional fragments, tizoxanide, dolutegravir, arbidol, and bictegravir were selected for interaction analysis according to the binding energy between small molecules and the S protein and the position of the binding site on the S protein.

**Table 1:** The highest binding energy of drug molecules with the S protein in molecular docking simulations.

Drug and drug treatment	S-binding energy (kcal/mol)
tizoxanide	-9.38
dolutegravir	-8.96
arbidol	-8.86
bictegravir	-8.72
oseltamivir carboxylate	-7.66
hydroxychloroquine	-7.50
chloroquine	-7.48
saquinavir	-6.72
darunavir	-5.56
favipiravir-4-ribofuranosyl-5’-triphosphate	-5.56
favipiravir-4-ribofuranosyl-5’- monophosphate	-5.40
lopinavir	-5.17
ribavirin 5’-monophosphate	-5.15
remdesivir triphosphate	-4.57

#### 3.1.2. Simulation analysis of tizoxanide and the S protein

**Fig 2:**
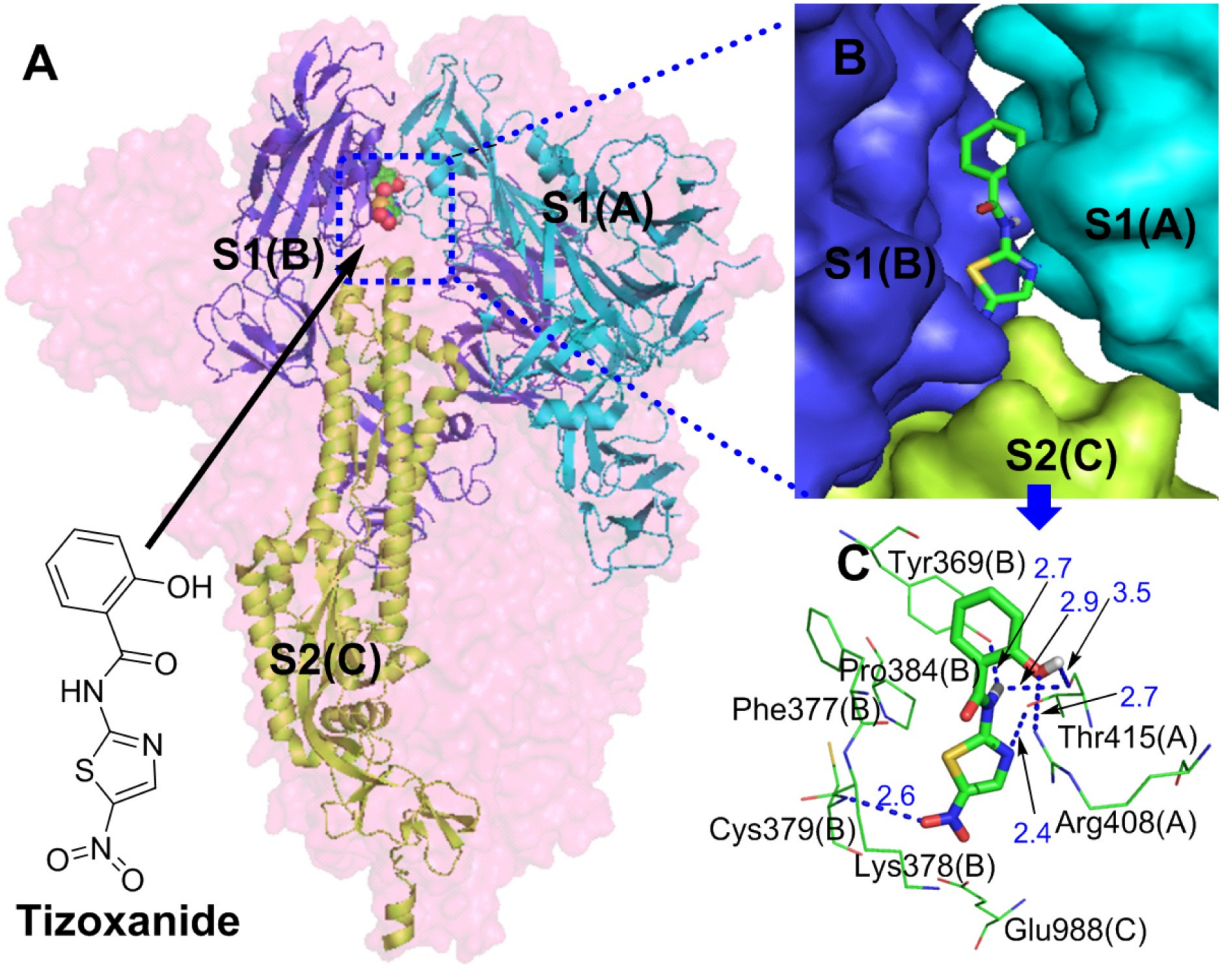
**(A)** Binding mode of the interaction of tizoxanide with the S protein. The S protein trimer is shown as a transparent red surface, S1(A), S1(B), and S2(C) are shown as cyan, blue, and yellow-green cartoons, respectively, and tizoxanide is shown as green spheres. **(B)** Binding mode of tizoxanide with the S protein. S1(A), S1(B), and S2(C) are shown as cyan, blue, and yellow-green surfaces, respectively, and tizoxanide is shown as green sticks. **(C)** The key residues that may form potential interactions with tizoxanide (green sticks) and polar interactions are indicated by blue dotted lines.

Fig 2A and 2B shows that tizoxanide binds at the junction of S1(A), S1(B) and S2(C) and simultaneously interacts with them. According to Table 1, the binding energy of tizoxanide is the highest (S-binding energy = -9.38 kcal/mol) among the analyzed small molecules, and there are three identical binding sites in each trimer of the S protein.

Fig 2C shows the interactions between tizoxanide and eight amino acid residues in S1(A), S1(B) and S2(C), of which Arg408(A), Thr415(A) and Gly416(A) are in the RBD of S1(A), Tyr369(B), Phe377(B), Lys378(B) and Cys379(B) are in the RBD of S1(B), and Glu988(C) is in the CH of S2(C) [26]. Interestingly, Cys379(B) in S1(B) is located in the core of the RBD, and the disulfide bond formed with its participation can stabilize the RBD [32]. Strong hydrogen bond networks and Van Der Waals forces (S1 Table) play important roles in the interactions between tizoxanide and S1(A), S1(C) and S2(B). Polar groups or atoms contained in tizoxanide are the basis of the hydrogen bond network: (1) a phenolic hydroxyl group interacts with the guanidine group of Arg408(A) and the carbonyl group of Thr415(A) through hydrogen bonds; (2) the NH of the amide group forms double hydrogen bonds with the phenolic hydroxyl group of Tyr369(B) and the carbonyl group of Thr415(A); (3) the N atom of the thiazole ring interacts with the hydroxyl group of Thr415(A) through a hydrogen bond; and (4) the strongly polar nitro group interacts with Cys379(B) through a hydrogen bond. Van Der Waals forces are formed between tizoxanide and Phe377(B), Lys378(B), and Glu988(C). In addition to the hydrogen bonds and Van Der Waals forces, the thiazole ring of tizoxanide also forms pi-alkyl hydrophobic interactions with Pro384(B). From the binding sites and interactions between tizoxanide and the S protein, tizoxanide simultaneously interacts with the RBD of S1(A) and S1(B) and the CH of S2(C) and not only stabilizes the RBD and prevents the binding between the RBD and ACE2 but also inhibits the fusion of S2 with host cells. Notably, the nitro group of tizoxanide interacts with Cys379(B) through a hydrogen bond, which may affect the disulfide bond formed by the participation of Cys379(B) in the RBD core, thereby affecting the stability of the RBD and its binding to ACE2. In the discovery of anti-SARS-CoV-2 drugs, some small molecules were found to interact with multiple functional fragments of the S protein simultaneously to inhibit S-mediated cell fusion; for example, nelfinavir interacts with HR1 and FP of the S protein through hydrogen bond networks and Van Der Waals forces to display an anti-SARS-CoV-2 effect [33]. In this study, by binding the S1 (RBD of S1(A) and S1(B)) and S2 (CH of S2(C)) subunits together through interactions such as hydrogen bond networks and Van Der Waals forces, tizoxanide may change the metastable conformation of the S protein before fusion into a stable conformation, resulting in S protein loss of function.

#### 3.1.3. Simulation analysis of bictegravir, dolutegravir and the S protein

**Fig 3:**
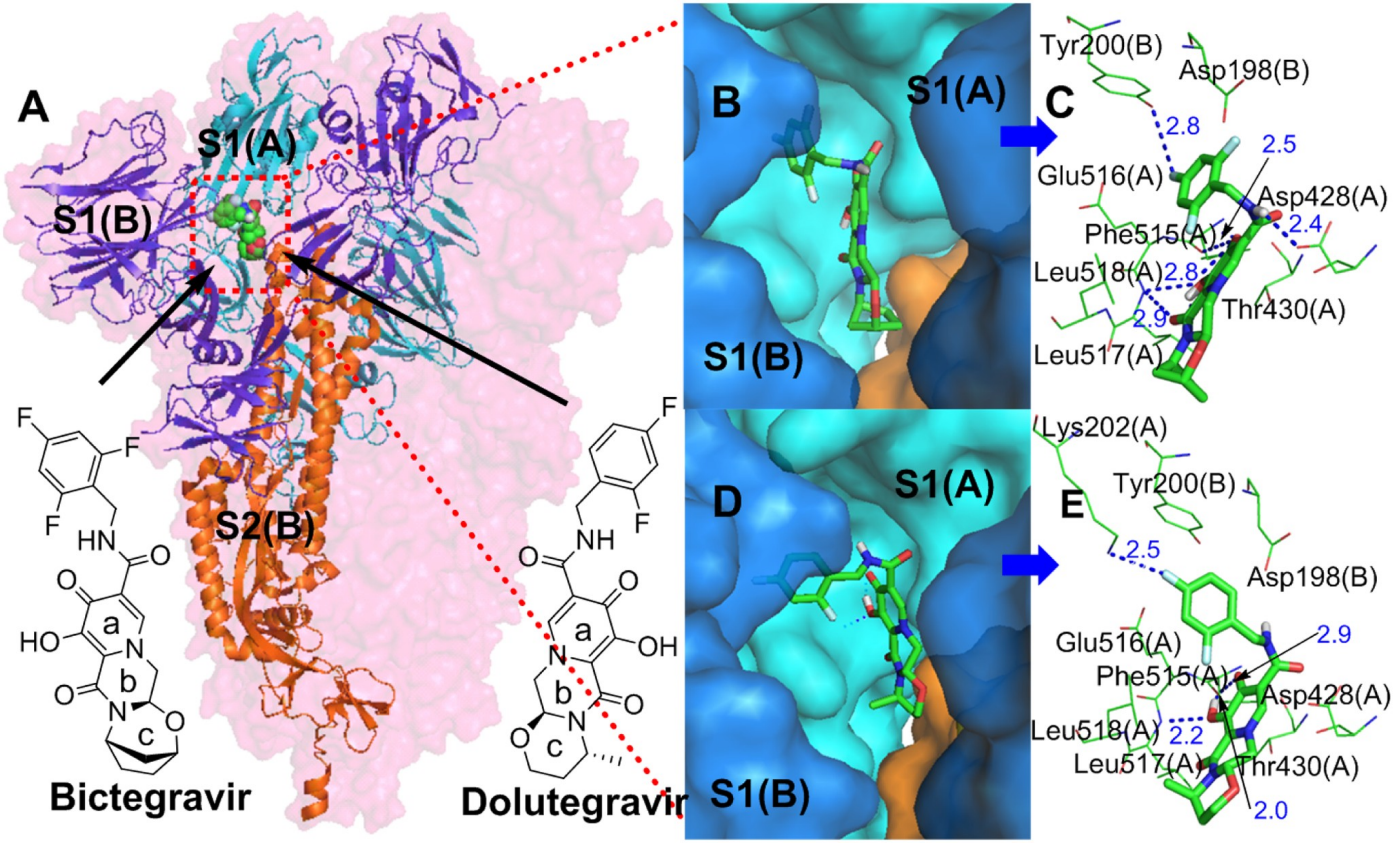
**(A)** Binding mode of the interactions of bictegravir and dolutegravir with the S protein. The S protein trimer is shown as a transparent red surface, S1(A), S1(B), and S2(B) are shown as cyan, blue, and orange cartoons, respectively, and bictegravir and dolutegravir are shown as green spheres. **(B)** and **(D)** Binding mode of bictegravir and dolutegravir (green) with the S protein. S1(A) and S1(B) are shown as cyan and blue surfaces, and bictegravir and dolutegravir are shown as green sticks. **(C)** and **(E)** The key residues that may form potential interactions with bictegravir and dolutegravir (green sticks) and polar interactions are indicated by blue dotted lines.

The structure of bictegravir is similar to that of dolutegravir. According to the simulation results, they both have a very good interaction with the S protein (dolutegravir, S-binding energy = -8.96 kcal/mol, bictegravir, S-binding energy = -8.72 kcal/mol). Both of them bind the same site (Fig 3A, 3B and 3D) between the RBD of S1(A) and the NTD of S1(B) with a similar conformation [26], and simultaneously interact with two protein fragments. Each trimer of the S protein contains three identical binding sites. The interaction mode of bictegravir and dolutegravir with the sites can be divided into two types: polar and nonpolar. For bictegravir (Fig 3C), the oxygen atom of the carbonyl and hydroxyl group of the a- and b-rings and the NH of the amide separately form strong hydrogen bond networks with the amino group of Phe515(A), the hydroxyl group of Leu517(A), and the carboxyl group of Asp428(A), respectively. Interestingly, the fluorine atom at the 4-position of the trifluorophenyl group also forms a hydrogen bond with the amino group of Tyr200(B). In addition, the oxygen atoms of the carbonyl and hydroxyl group of the a- and b-rings also form an intramolecular hydrogen bond, generating a stable conformation in the a- and b-rings. Additionally, the a-ring interacts with Thr430(A) through a pi-donor hydrogen bond. In addition to hydrogen bonds, Van Der Waals forces (S1 Table) are formed between bictegravir and some amino acid residues, for example, Phe515(A), halogen bonds are formed between the trifluorophenyl group and Glu516(A) and Asp198(B), and pi-alkyl hydrophobic interactions are formed between the c-ring and Leu518(A), which all contribute importantly to the binding of bictegravir to the S protein. For dolutegravir (Fig 3E), the intermolecular hydrogen bonds formed with S1(A) and S1(B) have the following three characteristics: (1) the fluorine atom at the 4-position of the difluorophenyl group forms hydrogen bonds with the amino group of Tyr200(B); (2) the carbonyl and hydroxyl groups of the a-ring form hydrogen bond networks with the amino group of Phe515(A) and the hydroxyl group of Leu517(A), respectively; and (3) the a-ring interacts with Thr430(A) and Asp428(A) through pi-donor hydrogen bonds. However, intramolecular hydrogen bonds for dolutegravir are not found. In terms of nonpolar interactions, dolutegravir forms Van Der Waals forces (S1 Table) and pi-alkyl hydrophobic interactions with Glu516(A) and Leu518(A), respectively.

The above analysis shows that bictegravir and dolutegravir embedded between the RBD of S1(A) and the NTD of S1(B) of the S protein trimer form close relationships with S1(A) and S1(B) through polar and nonpolar interactions. These interactions could limit the conformational change in the RBD during the invasion of SARS-CoV-2 into host cells, thus blocking the binding of the RBD and ACE2 and preventing viral invasion.

#### 3.1.4. Simulation analysis of arbidol and the S protein

**Fig 4:**
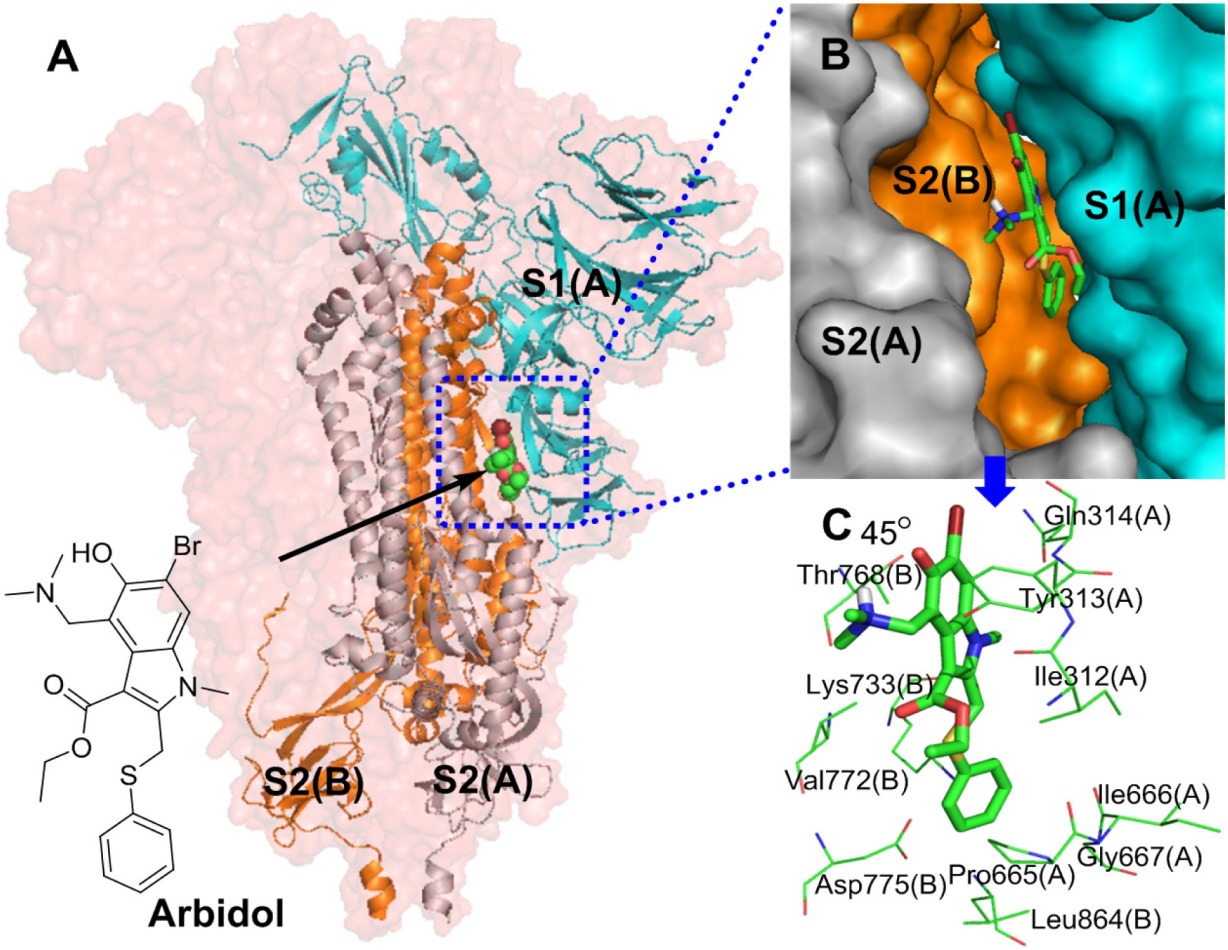
**(A)** Binding mode of the interaction of arbidol with the S protein. The S protein trimer is shown as a transparent red surface; S1(A), S2(A), and S2(B) are shown as cyan cartoons; and arbidol is shown as a green sphere. **(B)** Binding mode of arbidol (green) with the S protein. S1(A), S2(A), and S2(B) are shown as cyan surfaces, and arbidol is shown as a green stick. **(C)** The key residues that may form potential interactions with arbidol (green stick) and polar interactions are indicated by blue dotted lines.

Simulation results show that arbidol and the S protein have good interactions, with a binding energy of -8.86 kcal/mol. As shown in Fig 4A and 4B, the most important conformational binding sites of arbidol are located in the hydrophobic cavity between S1(A) and S2(B) of the stem region of the S protein, and each trimer of the S protein has three identical binding sites with arbidol. The indole ring and phenylthiomethyl group, the core skeleton, are located inside the hydrophobic cavity, and the dimethylamine and ethyl carboxylate groups on the indolyl group face the outside of the hydrophobic cavity.

Fig 4C shows the interactions between arbidol and S1(A) and S2(B). Among the amino acid residues that interact with arbidol are Gly311(A), Ile312(A), Tyr313(A), Gln314(A), Pro665(A), Ile666(A) and Gly667(A) in S1(A) and Thr768(B), Val772(B), Asp775(B) and Leu864(B) in S2(B). The interactions between arbidol and S1(A) and S2(B) are mostly driven by nonpolar interactions. The indolyl group and the substituent groups of arbidol form multiple interactions with multiple amino acid residues. The pyrrole ring and ethyl carboxylate substituents of the indolyl group form pi-alkyl hydrophobic interactions with Ile312(A). The benzene ring of the indolyl group simultaneously forms a pi-sigma hydrophobic interaction with Tyr313(A) and Thr768(B), and the N-methyl group of the pyrrole ring also forms a hydrophobic interaction with Thr768(B). Moreover, the benzene ring of the indolyl group also forms a pi-donor hydrogen bond interaction with Gln314(A). The phenylthio group of arbidol is at the contact site of S1(A) and S2(B) and forms complex interactions with many surrounding amino acid residues. Specifically, the benzene ring of the phenylthio group forms amide-π stacked, pi-cation, pi-anion, and pi-alkyl interactions with Pro665(A), Lys733(B), Asp775(B) and Leu864(B), respectively. A sulfur atom interacts with Ile312(A), Pro665(A) and Val772(B) via hydrophobic interactions. In addition to the interactions discussed above, arbidol forms Van Der Waals (S1 Table) interactions with Val772(B), Ile 666(A) and Gly667(A). In summary, through the above interactions, arbidol is stably embedded between S1(A) and S2(B) and binds S1(A) to S2(B), stabilizing the structure of the S protein. Arbidol can thus block the shedding of the S1 subunit and inhibit the ability of S2 to exert a fusion function when SARS-CoV-2 invades host cells; therefore, SARS-CoV-2 cannot invade host cells.

### 3.2. Molecular optimization and simulation analysis

#### 3.2.1. Tizoxanide optimization and simulation analysis

**Fig 5:**
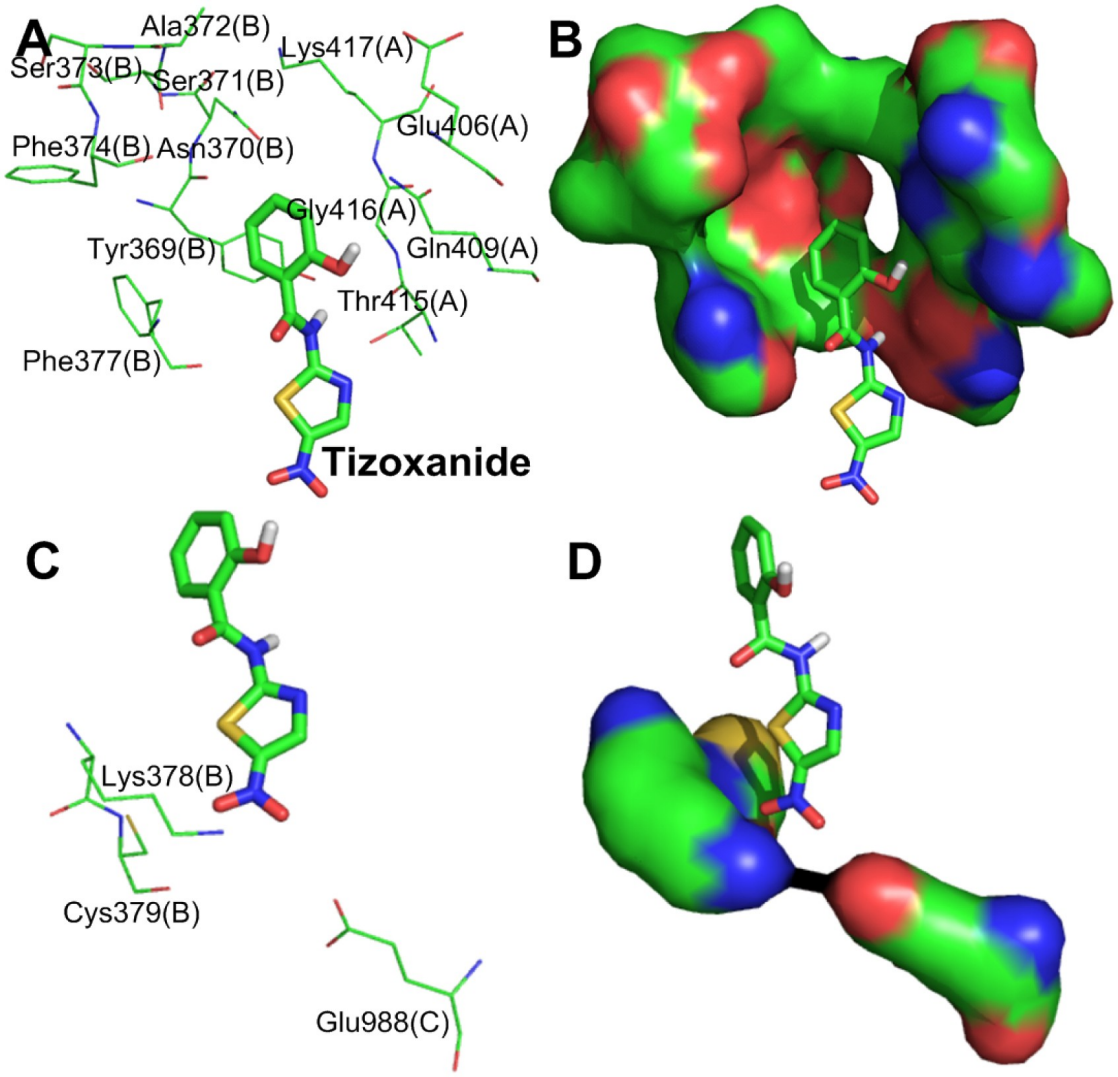
**(A)** and **(C)** The amino acid residues that surround the phenol group and nitro group of tizoxanide (green). **(B)** and **(D)** The amino acid residues surrounding the phenol and nitro groups are shown as green surfaces.

The molecular docking results show that tizoxanide and the S protein have the highest binding energy and that the location of tizoxanide is also critical. However, in terms of the interactions between tizoxanide and S1(A), S1(B), and S2(C) and the surrounding amino acid environment, tizoxanide also has a large optimization space. The amino acid residues surrounding the phenolic group of tizoxanide contain a large number of polar groups (the carboxyl group of Glu406(A), the amino group of Lys417(A), the phenolic hydroxyl group of Tyr369(B), the hydroxyl group of Ser371(B), and amide groups in various amino acid residues) and many nonpolar groups (e.g., the 5-membered carbon chain of Lys417(A) and the benzene rings of Tyr369(B), Phe374(B), and Phe377(B)). In this region, both polar interactions, such as hydrogen bonds between polar molecular fragments and polar groups, and hydrophobic interactions, such as the interactions between hydrophobic groups (e.g., methyl group) and nonpolar groups, can be introduced. The nitro group of tizoxanide is adjacent to Lys378(B), Cys379(B) and Glu988(C), which all contain polar groups, and replacing the nitro group with a strong polar group is conducive to forming a strong interaction with the above three amino acid residues (Fig 5).

**Fig 6:**
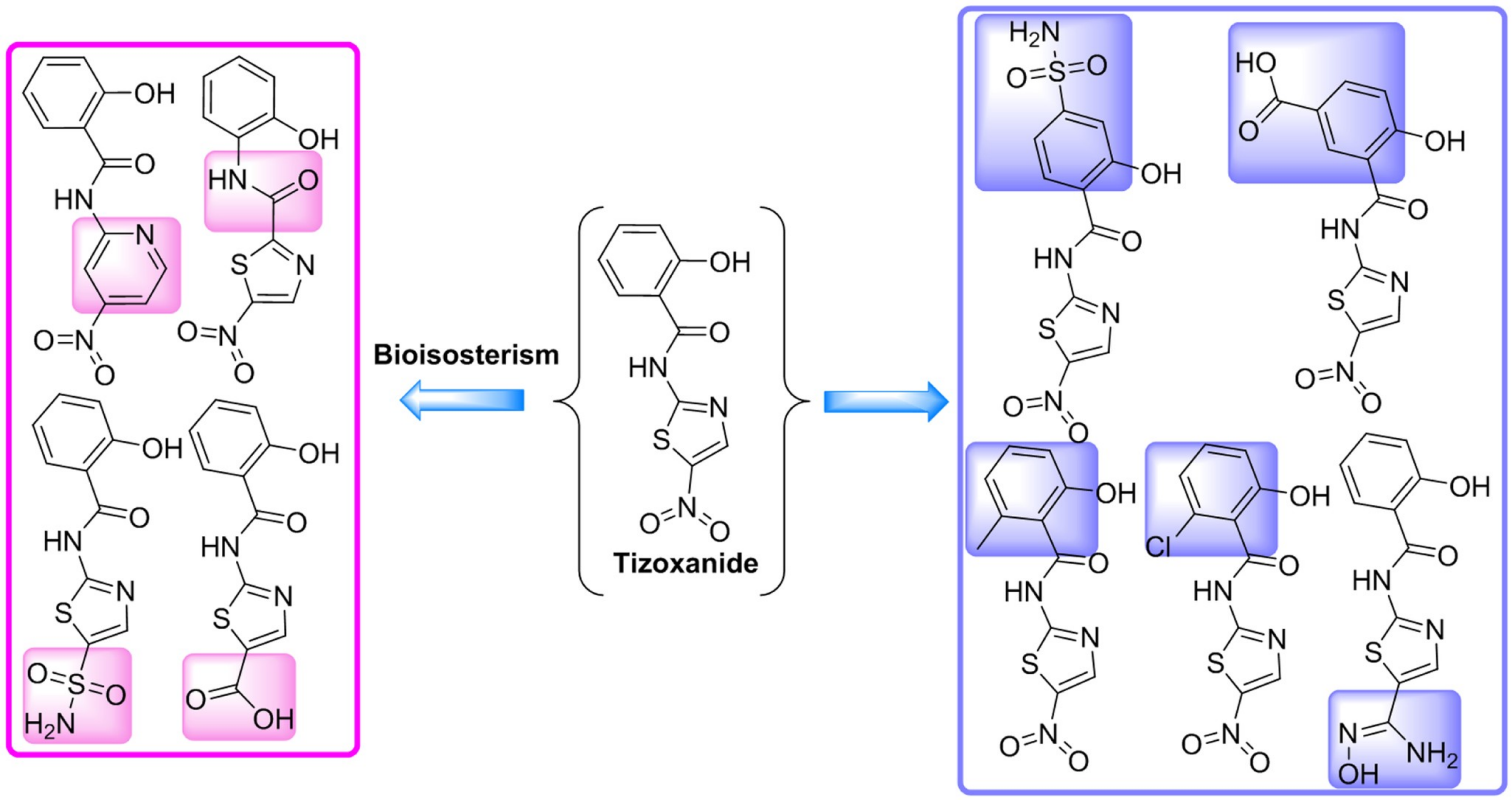
Strategy for further structural optimization of tizoxanide.

Based on the above analysis, which showed that the para positions of the amide group and phenolic hydroxyl group on the benzene ring of tizoxanide are adjacent to polar amino acid residue segments (Fig 5B), strong polar groups (carboxyl and sulfonamide groups) can be introduced by the molecular connection method at these two positions to form polar interactions with the surrounding amino acids. The ortho position of the amide group, which is also the interposition of the hydroxyl group, is close to the nonpolar segments (Fig 5B); therefore, a methyl group and chlorine atom can be introduced for validation analysis. The bioisosterism strategy can be used to replace the nitro group with sulfonamide, a carboxyl group, or a basic N-hydroxyindenyl group. In addition, for the amide group or thiophene ring, molecules with better binding ability may be obtained by inverting the amide group or replacing the thiophene ring with a pyridine ring via the bioisosterism strategy (the optimization strategy is shown in Fig 6).

**Fig 7:**
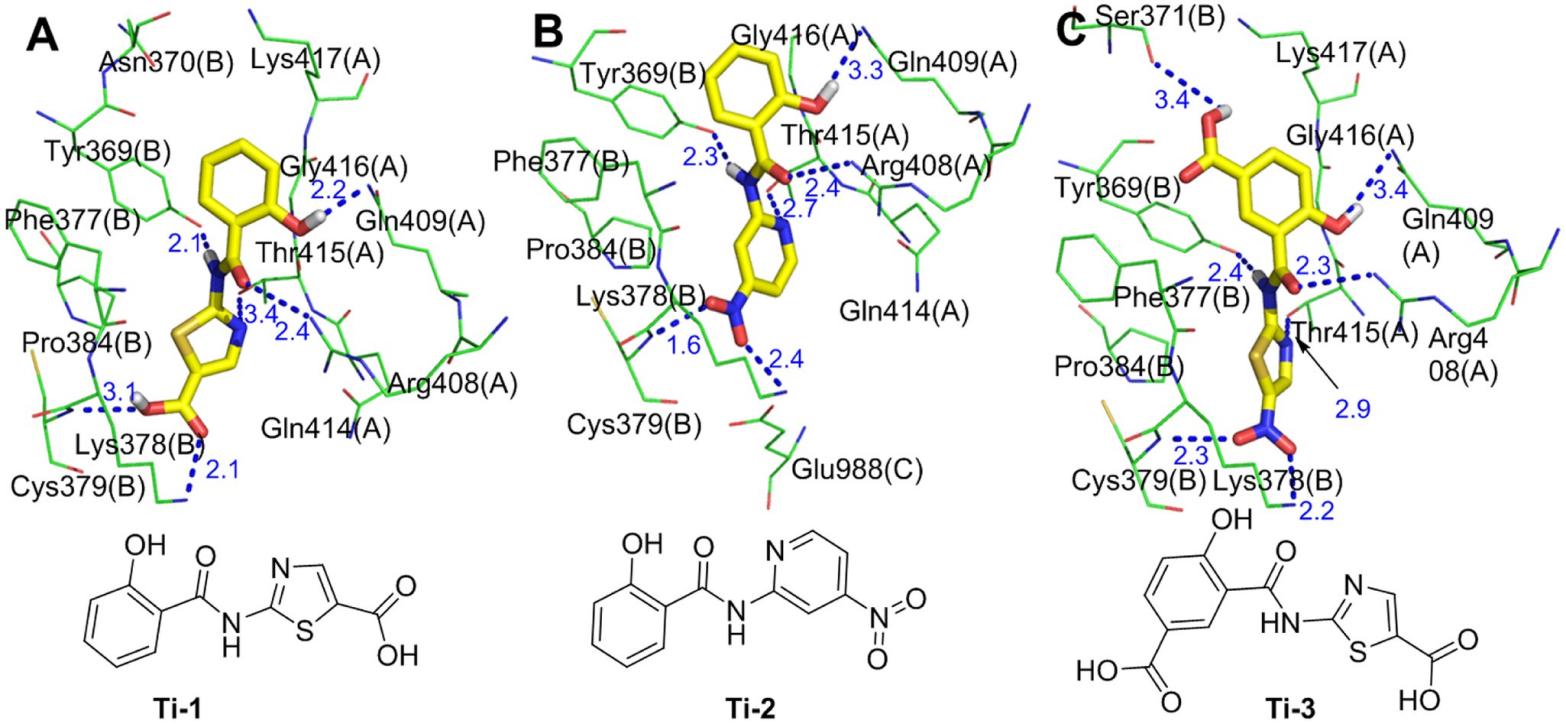
Molecular mode results for Ti-1(A), Ti-2(B), and Ti-3(C) with the S protein and the key residues that may form potential interactions with compounds Ti-1, Ti-2 and Ti-3.

The molecular simulation results show that for the optimized molecules **Ti-1** (the product in which the nitro group of tizoxanide is replaced with a carboxyl group), **Ti-2** (the product in which the thiazole ring of tizoxanide is replaced with a pyridine ring), and **Ti-3** (the product in which the phenolic hydroxyl group of tizoxanide is para-linked to a carboxyl group), each conformation has a very good binding affinity with the S protein (binding energies of -9.67 kcal/mol, -10.94 kcal/mol, and -10.49 kcal/mol, respectively). Fig 7 shows the interactions between the major conformations of **Ti-1**, **Ti-2** and **Ti-3** and the S protein, in which **Ti-1** and **Ti-3** interact with S1(A) and S1(B) and **Ti-2** interacts with S1(A), S1(B) and S2(C). Notably, the above three molecules are similar to tizoxanide and interact with the surrounding amino acid residues through strong hydrogen bond networks. There are similarities and differences among these hydrogen bond interactions. The similarities are as follows: the phenolic hydroxyl group and the oxygen atom of the carbonyl and NH sites of the amide groups of the three molecules form hydrogen bonds with Gln409(A), Arg408(A) and Tyr369(B), respectively; and the differences are as follows: (1) **Ti-1** forms double hydrogen bonds with Lys378(B) and Cys379(B) through the carboxyl group, while **Ti-2** and **Ti-3** form double hydrogen bonds with Lys378(B) and Cys379(B) via a nitro group; (2) **Ti-1** and **Ti-3** form a hydrogen bond interaction with Thr415(A) through the N atom of the thiazole ring, while **Ti-2** interacts with Thr415(A) through a hydrogen bond formed by the N atom on the pyridine ring; and (3) the newly introduced carboxyl group on the benzene ring of **Ti-3** also forms a new hydrogen bond with Ser371(B), and this hydrogen bond does not exist in **Ti-1** and **Ti-2**. In addition, the benzene rings of **Ti-1** and **Ti-2** separately interact with Lys417(A) and Tyr369(B) by forming pi-donor hydrogen bonds, but no such atypical hydrogen bond is observed in **Ti-3**. **Ti-1**, **Ti-2**, and **Ti-3** form Van Der Waals (S1 Table) interactions with the S protein through four amino acid residues (Gln414(A), Gly416(A), Asn370(B) and Phe377(B)), three amino acid residues (Gln414(A), Phe377(B) and Glu988(C)), and two amino acid residues (Phe377(B) and Pro384(B)), respectively. In addition to hydrogen bonds and Van Der Waals forces, **Ti-1** and **Ti-2** form pi-alkyl interactions with Pro384(B) via a thiazole ring and pyridine ring, respectively.

The above analysis shows that compared with that of tizoxanide, the binding energy of the optimized molecules (**Ti-1**, **Ti-2**, and **Ti-3**) is improved, and in terms of interaction, they have not only the same types of interactions with more amino acid residues but also new types of interactions. For example, **Ti-1** and **Ti-2** form pi-donor hydrogen bonds with the S protein, and this interaction is not observed in tizoxanide. In addition, **Ti-1, Ti-2** and **Ti-3** still form hydrogen bond interactions with Cys379(B), and **Ti-2** also interacts with the CH of S2. Overall, **Ti-1**, **Ti-2** and **Ti-3** bind to the S protein with stronger affinity.

#### 3.2.2. Optimization of bictegravir and dolutegravir and simulation analysis

Despite the high binding energy of bictegravir and dolutegravir with the S protein (Fig 3), the c-rings of both compounds extend beyond the binding site and interacts only with Leu518(A) to form alkyl hydrophobic interactions, indicating that at this binding site, a c-ring with a large volume is not the optimal molecular fragment. Similarly, the trifluorophenyl and difluorophenyl regions are relatively narrow and cannot sufficiently accommodate these two molecular fragments and form strong interactions. Therefore, it is necessary to perform reasonable structure-based optimization based on their conformations in the S protein and their interactions with S1(A) and S1(B).

**Fig 8:**
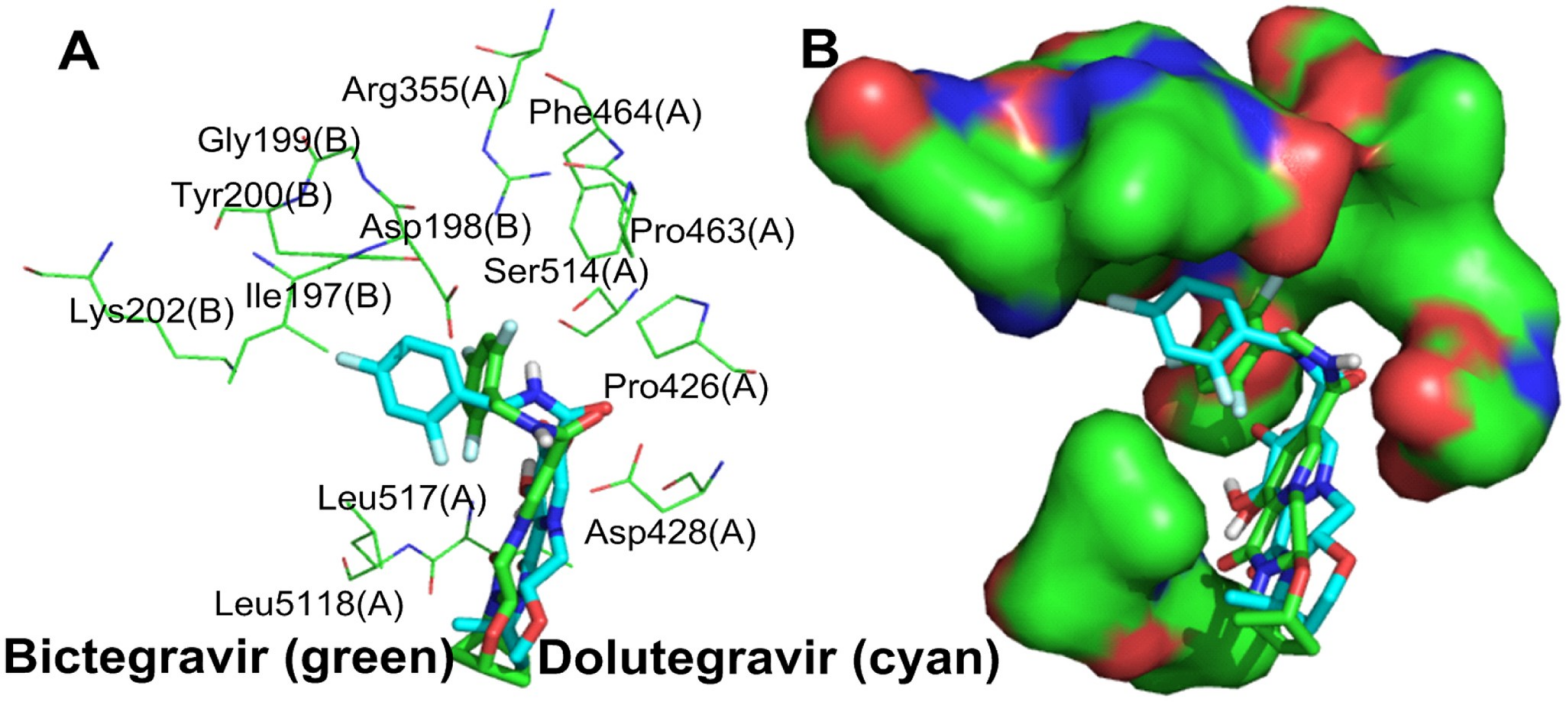
**(A)** The amino acid residues that surround the trifluorophenyl and difluorophenyl moieties of bictegravir (green) and dolutegravir (blue-green). **(B)** The amino acid residues surrounding the trifluorophenyl and difluorophenyl moieties are shown as green surfaces.

As shown in Fig 8A and 8B, on the one hand, only two hydrophobic amino acid residues, Leu517(A) and Leu518(A), are distributed around the c-ring of bictegravir and dolutegravir, and hydrophobic molecular fragments with small volumes may be more suitable in this region; on the other hand, there are both hydrophilic and hydrophobic amino acid residues surrounding the trifluorophenyl and difluorophenyl groups. The guanidine group of Arg355(A), the carboxyl group of Asp428(A), the hydroxyl group of Ser514(A), the carboxyl group of Asp198(B), the phenolic hydroxyl group of Tyr200(B) and the amino group of Lys202(B) provide a good environment for the introduction of hydrophilic molecular fragments, and hydrophobic amino acid fragments of Pro426(A), Pro463(A), Phe464(A), Ile197(B) and Tyr200(B) provide favorable conditions for introducing hydrophobic molecular fragments. The above analysis is the basis for the rational optimization of bictegravir and dolutegravir.

**Fig 9:**
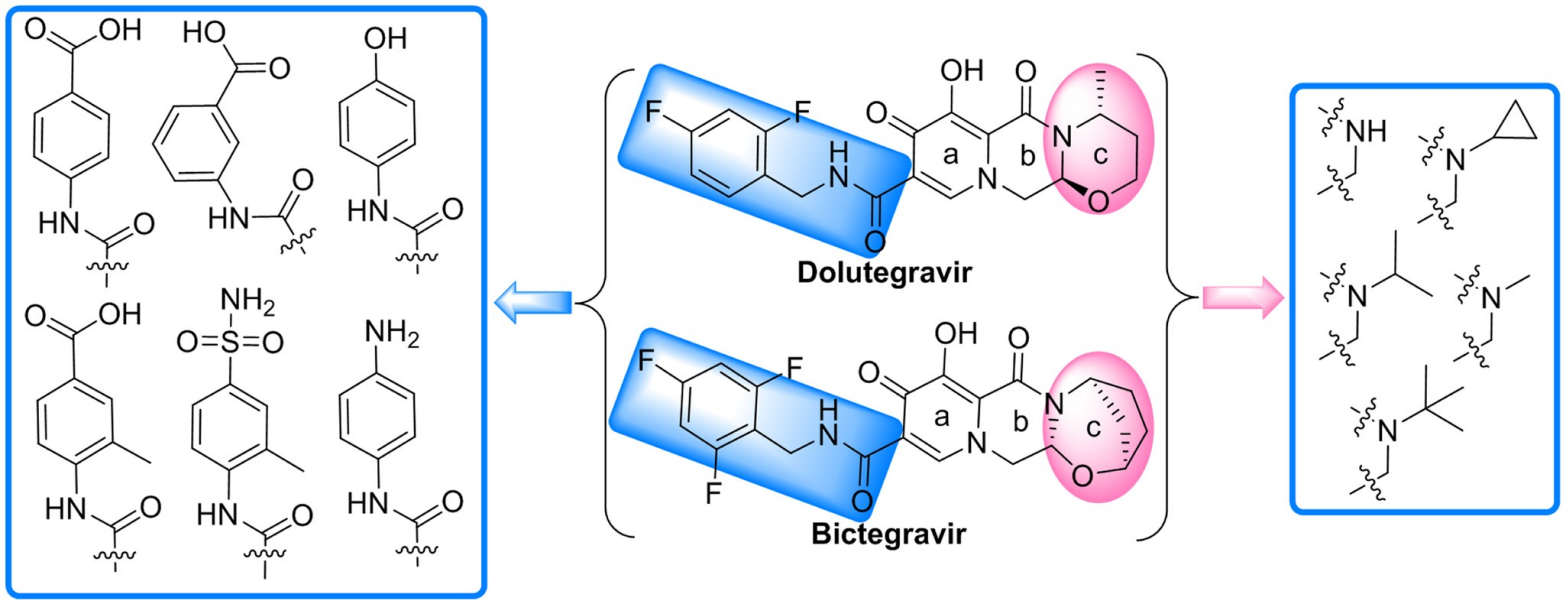
Strategy for further structural optimization of bictegravir and dolutegravir.

Based on the above analysis, the optimization of bictegravir and dolutegravir mainly focuses on two aspects to obtain small molecules that bind better to the S protein. First, the c-ring is changed to a hydrophobic molecular fragment with a small volume. Specifically, the c-ring is directly removed, or hydrophobic fragments (methyl, isopropyl, cyclopropyl, or tert-butyl) smaller than the c-ring are subsequently introduced to the N atom of the b-ring to form hydrophobic interactions with Leu517(A) and Leu518(A). Second, the linker between the a-ring and the benzene ring is replaced with an amide, thereby shortening the length of the molecule to accommodate the binding site space; the trifluorophenyl or difluorophenyl groups are then changed to molecular fragments containing both hydrophilic groups, such as carboxyl, sulfonamide, phenolic hydroxyl and amino groups, and a hydrophobic benzene ring or methyl benzene ring, ensuring not only that the hydrophilic groups in the segment form hydrophilic interactions with the amino acid residues containing hydrophilic segments but also that the benzene ring or methyl benzene ring forms hydrophobic interactions with the amino acid residues containing hydrophobic fragments, as shown in Fig 9.

**Fig 10:**
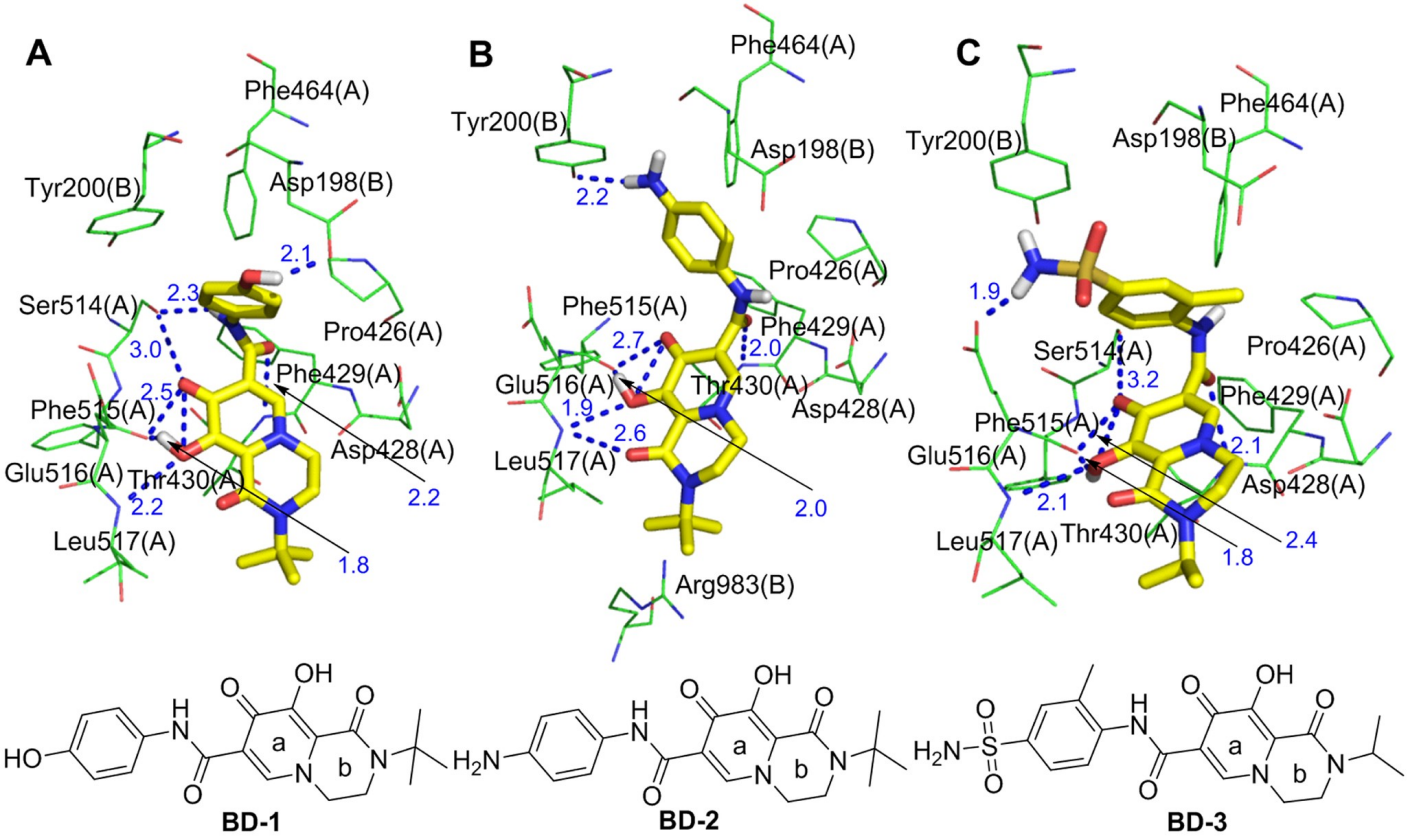
Molecular mode results for BD-1(A), BD-2(B), and BD-3(C) with the S protein and the key residues that may form potential interactions with compounds BD-1, BD-2, and BD-3.

By comparing the interactions and binding energies of the optimized molecules with the S protein and overlapping the optimized molecules with the bictegravir and dolutegravir conformations, the molecules **BD-1**, **BD-2** and **BD-3** were optimized to have conformations with strong interactions with the S protein (S-binding energy = -11.31 kcal/mol, -11.09 kcal/mol, and -10.85 kcal/mol, respectively). As shown in Fig 10, with the exception of intramolecular hydrogen bonds, **BD-1**, **BD-2** and **BD-3** all form dense and powerful hydrogen bond networks with S1(A) and S1(B). (1) **BD-1** separately forms a sevenfold hydrogen bond network with five amino acid residues (Ser514(A), Phe515(A), Leu517(A), Thr430(A), and Asp198(B)) through the oxygen atoms of the carbonyl and hydroxyl groups in the a-ring, the NH site of the amide groups, and the phenolic hydroxyl group. (2) **BD-2** separately forms a sixfold hydrogen bond network with four amino acid residues (Phe515(A), Leu517(A), Thr430(A) and Tyr200(B)) through the oxygen atoms of the carbonyl and hydroxyl groups in the a- and b-rings, oxygen atoms of the carbonyl groups in the amide group, and the anilino groups. (3) **BD-3** separately forms a sixfold hydrogen bond network with five amino acid residues (Ser514(A), Phe515(A), Leu517(A), Thr430(A) and Glu516(A)) through the oxygen atoms of the carbonyl and hydroxyl groups in the a-ring, the oxygen atoms of the amide groups, and the sulfonamide group. In summary, **BD-1**, **BD-2** and **BD-3** form more hydrogen bonds with the S protein than do bictegravir and dolutegravir. In addition, **BD-1**, **BD-2** and **BD-3** can form pi-donor hydrogen bond interactions with Asp428(A), Phe429(A) and Thr430(A), and the newly introduced sulfonamide group in **BD-3** can also form a pi-donor hydrogen bond interaction with Tyr200(B). Furthermore, **BD-1** and **BD-2** can form hydrophobic interactions with Leu517(A) through the newly introduced tert-butyl group. In addition, **BD-2** forms a hydrophobic interaction with Pro426(A). **BD-3** forms hydrophobic interactions with Pro426(A) and Tyr200(B). Bictegravir and dolutegravir can form hydrophobic interactions only with an amino acid residue (Leu518(A)).

**BD-1**, **BD-2** and **BD-3** can form Van Der Waals (S1 Table) interactions with the amino acid residues of S1(A) and S1(B), more so than bictegravir (Phe515(A)) and dolutegravir (Glu516(A)). Interestingly, Van Der Waals interactions between **BD-2** and Arg983(B) were not observed with bictegravir, dolutegravir, **BD-1** or **BD-3**. Arg983(B) belongs to the amino acid residue of the HR1 fragment in S2(B), and HR1 plays an essential biological function in the membrane fusion stage of the **SARS-CoV-2** life cycle [24]. These results indicate that **BD-2** can inhibit the conformational changes not only in the RBD in S1 but also in S2 and can affect the fusion of SARS-CoV-2 with host cell invasion, which is a new discovery regarding the interactions between optimized bictegravir and dolutegravir molecules and the S protein. In summary, the binding affinities of **BD-1**, **BD-2** and **BD-3** with the S protein are stronger than those of bictegravir and dolutegravir.

#### 3.2.3. Optimization of arbidol and simulation analysis

Arbidol and S1(A) and S2(B) can form a relatively good interaction; however, some shortcomings remain in the relative positional relationships and interactions between arbidol and S1(A) and S2(B). On the one hand, the dimethylamino group attached to the indolyl group extends to the outside of the hydrophobic cavity formed by S1(A) and S2(B) (Fig 4) and does not form any interactions with any amino acid residues. In addition, the Br atom and phenolic hydroxyl group on the benzene ring of indolyl do not interact with any amino acid residues. The above three groups become potential molecular fragments that can be deleted or replaced. On the other hand, the N-methyl group of the pyrrole ring and phenylthio group are adjacent to an unoccupied hydrophobic region, which provides space for structure-based molecular optimization. Therefore, it is necessary to carry out molecular optimization of arbidol so that optimized arbidol derivatives can better interact with S1(A) and S2(B).

**Fig 11:**
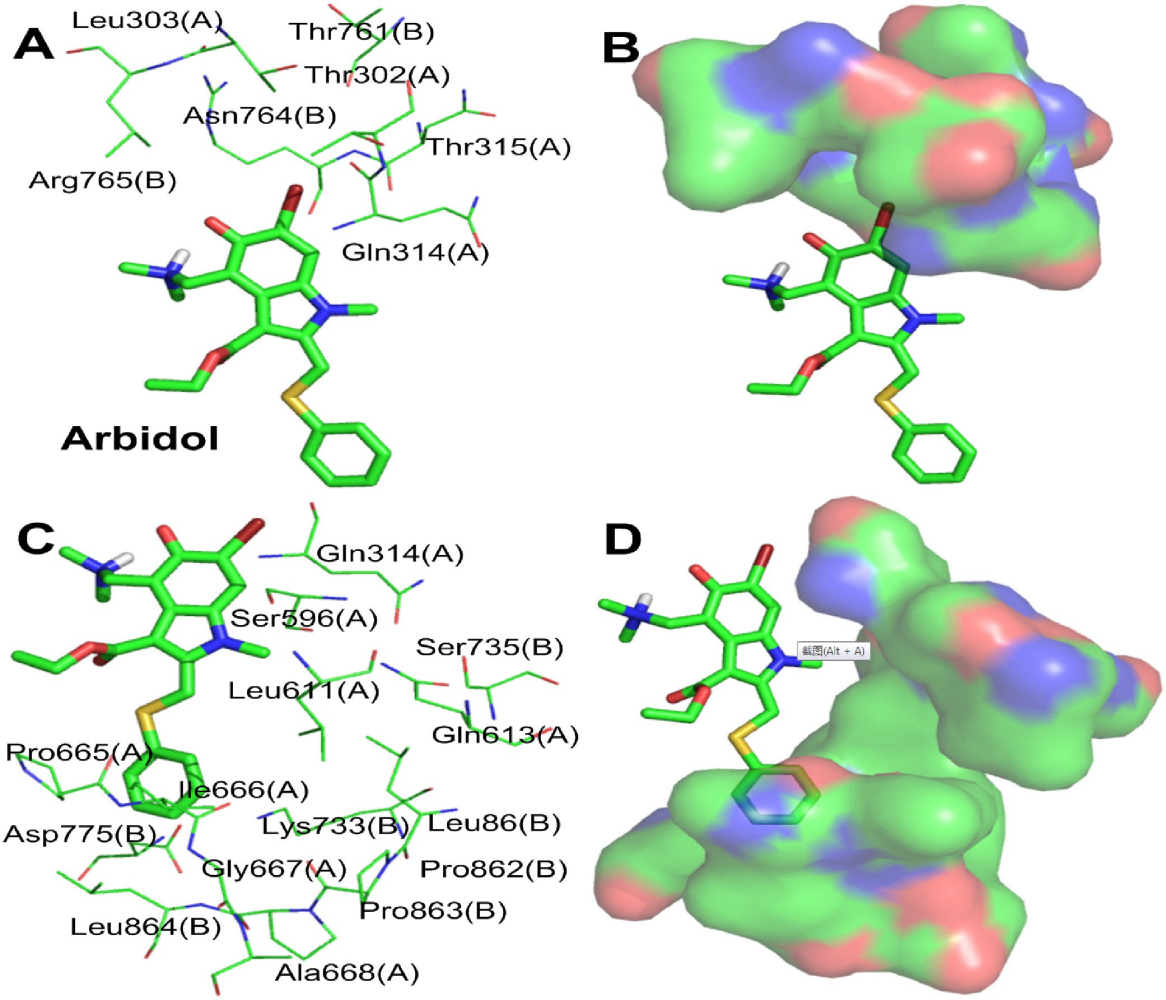
**(A)** The polar residues that surround the Br of arbidol (green). **(B)** The H-binding region consists of polar residues, which are shown as a transparent surface. **(C)** The hydrophobic residues that surround the N-methyl group. **(D)** The arbidol–hydrophobic residue complex; the hydrophobic residues are shown as a transparent surface.

By analyzing the binding modes and interactions between arbidol and S1(A) and S2(B), with the exception of Leu303(A), the Br atom is surrounded by hydrophilic amino acid residues (Thr302(A), Gln314(A), Thr315(A), Thr761(B), Asn764(B) and Arg765(B)), and these amino acid residues together form a hydrogen bond region (Fig 11A and 11B). Therefore, it is possible to replace the Br atom with an acidic or basic group to form hydrogen bonds with the strongly polar amino acid residues in the hydrogen bond region. The N-methyl group of the pyrrole ring and phenylthio group are surrounded by amino acid residues S1(A) and S2(B) that are hydrophobic or contain hydrophobic fragments (Fig 11C), and these amino acid residues form a large hydrophobic region (Fig 11D). This region can be used to enhance the hydrophobic interaction between the molecule and the region by introducing appropriate hydrophobic groups on the N of the pyrrole ring or S atom by the molecular connection method.

**Fig 12:**
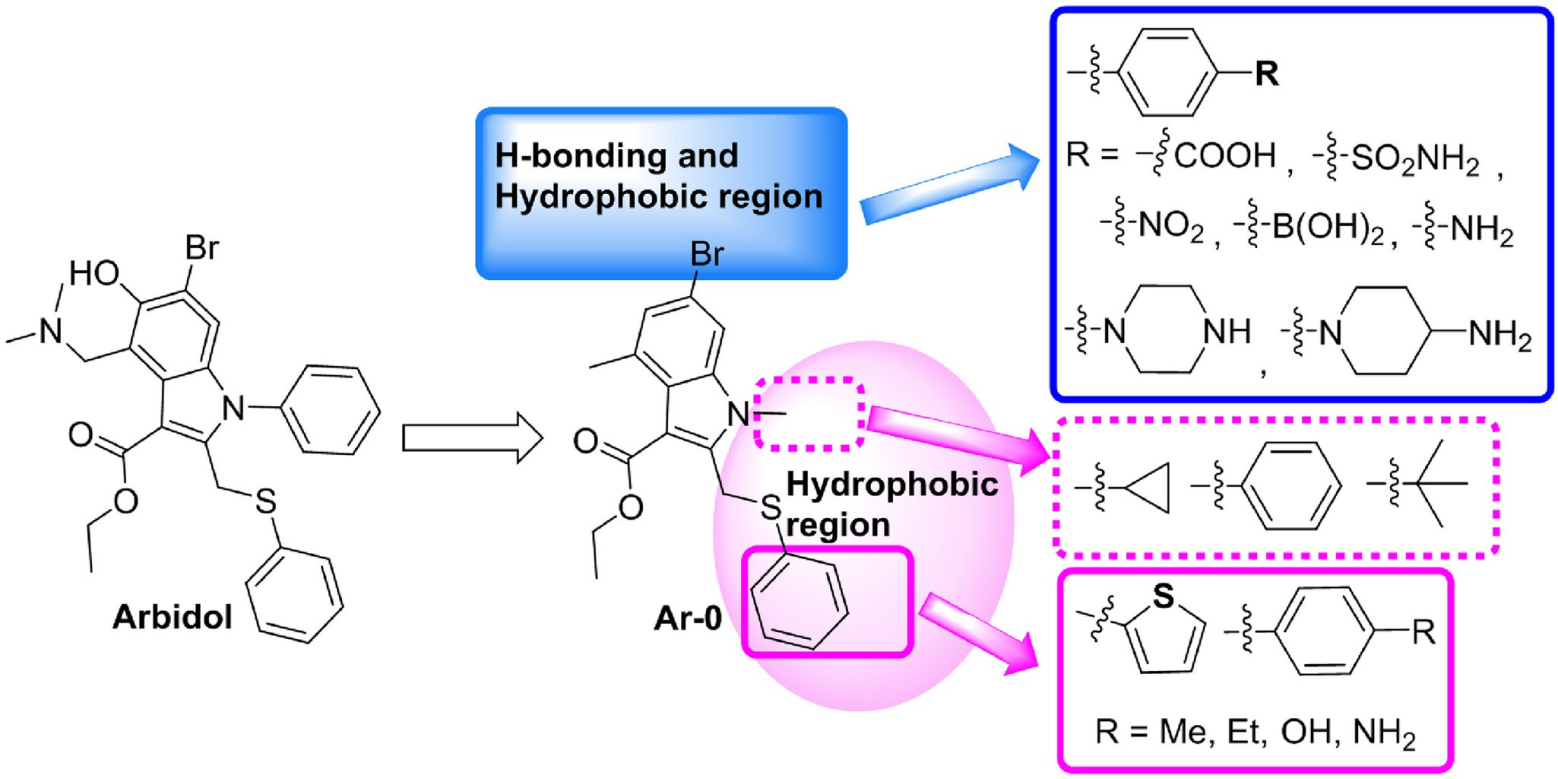
Strategy for further structural optimization of arbidol.

Our strategy here was to explore and optimize the Br, N,N-dimethylamino and benzyl sulfides of arbidol and identify structure-interaction relationships that could be utilized to improve the binding affinity of arbidol derivatives and the S protein. To this end, the dimethylamino and phenolic hydroxyl groups of arbidol were first deleted to obtain the molecule **Ar-0**. Subsequently, three series of new arbidol derivatives were designed based on **Ar-0**. In series I, acidic groups (*p*-benzoic acid, *p*-benzene sulfonamide, *p*-nitro or *p*-boronic acid) and basic groups (*p*-aniline, piperazinyl, or N-1-piperidine-4-amino) were used to replace the Br atom. In series II, a tert-butyl group, a cyclopropyl group or a phenyl group was attached to the N atom of the pyrrole ring. In series III, the benzene ring of the phenylthio group was replaced with thienyl, *p*-methylphenyl, *p*-ethylphenyl, *p*-hydroxyphenyl or *p*-aminophenyl using the bioisosterism strategy and molecular diversity rule (Fig 12). We were optimistic that these designs and optimizations would be fruitful.

**Fig 13:**
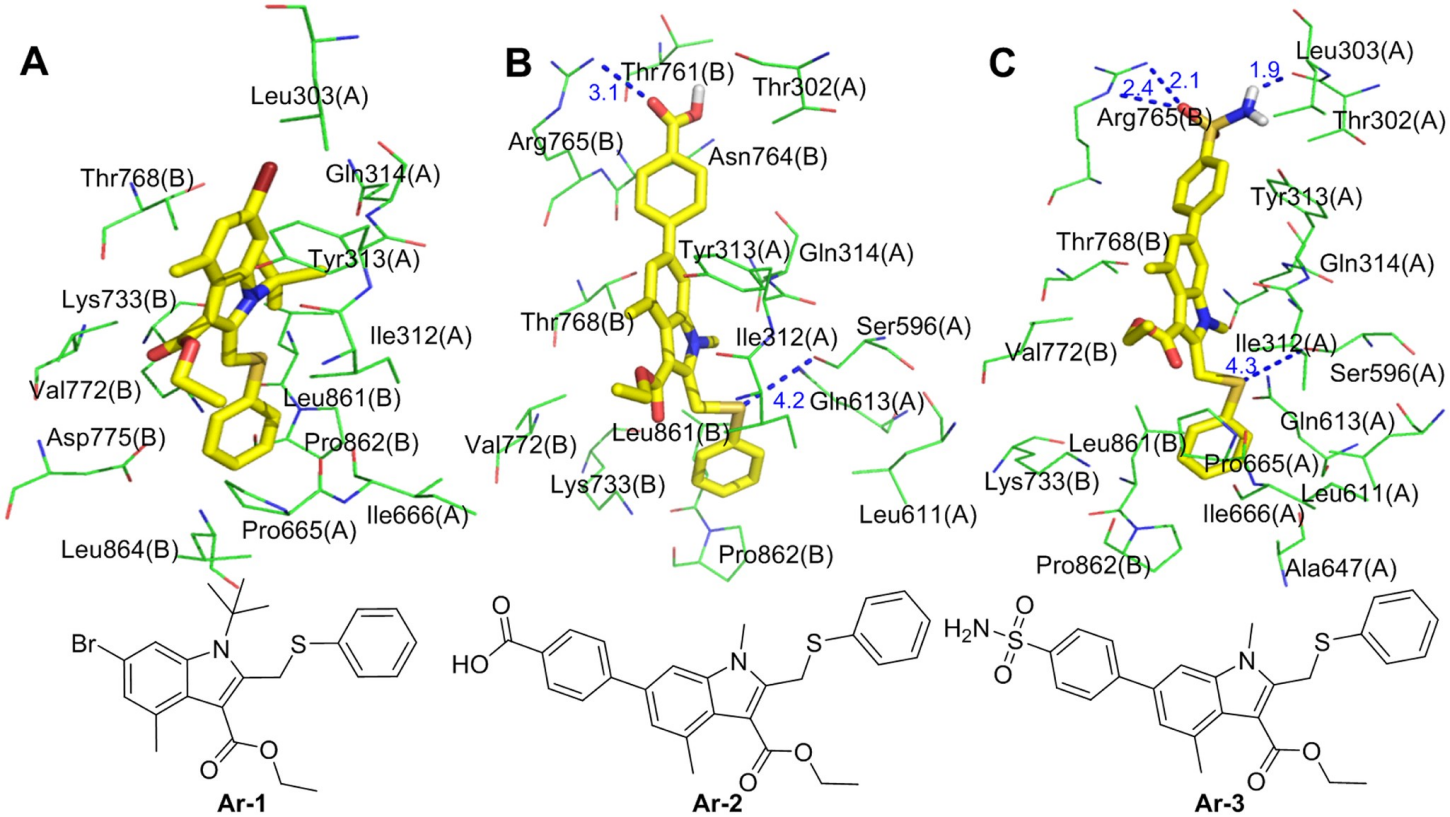
Molecular mode results for Ar-1 (A), Ar-2 (B), and Ar-3 (C) with the S protein and the key residues that may form potential interactions with compounds Ar-1, Ar-2, and Ar-3.

The molecular simulations found that one conformation each of **Ar-1**, **Ar-2** and **Ar-3** has a very high binding energy and good overlap with arbidol at the binding sites. **Ar-1** is the product in which the methyl group on the N atom of the **Ar-0** pyrrole ring is replaced with a tert-butyl group. The molecular simulation results showed that the binding energy of **Ar-1** and the S protein is -11.70 kcal/mol, which is much higher than that of arbidol with the S protein (Table 1). Compared with arbidol, the number of amino acid residues of S1(A) and S2(B) that form the nonpolar interactions with **Ar-1** increased (Leu303(A), Leu861(A) and Pro862(A)), and they form Van Der Waals (S1 Table) interactions with **Ar-1**. In addition, the methyl group on the benzene ring of the indolyl group forms a pi-alkyl hydrophobic interaction with Tyr313(A), and the newly introduced tert-butyl group forms hydrophobic interactions with Ile312(A), Tyr313(A), Gln314(A) and Thr768(B) (Fig 13A). The enhancement of these nonpolar interactions is reflected by the binding energy of **Ar-1** and the S protein, which also indicates that compared to arbidol, the binding affinity between **Ar-1** and the S protein is enhanced.

**Ar-2** is the product in which the Br atom of **Ar-0** is replaced with a 4-carboxyphenyl group, greatly improving the binding energy with the S protein. As shown in Fig 13B, the 4-carboxyphenyl group extends into the hydrogen bond and hydrophobic regions, in which the carboxyl group interacts with the guanidine group of Arg765(B) via a hydrogen bond; the 4-carboxyphenyl group also separately forms Van Der Waals forces (S1 Table) and pi-alkyl hydrophobic interactions with Thr302(A), Thr761(B), Asn764(B) and Arg765(B). The methyl group on the phenyl ring in the indolyl group also forms a pi-alkyl hydrophobic interaction with Tyr313(A). The interactions between the indole ring and its replaced ethyl carboxylate and N-methyl groups of **Ar-2** and the surrounding amino acid residues are almost the same as those of arbidol. Compared with that of arbidol, the phenylthio group of **Ar-2** undergoes a conformational change under the influence of the 4-carboxyphenyl group, and its interactions with S1(A) and S2(B) change accordingly. (1) The S atom of the phenylthio group interacts with the hydroxyl group of Ser596(A) via a hydrogen bond, and (2) the phenyl group not only interacts with Leu611(A), Gln613(A), Lys733(B) and Pro862(B) through Van Der Waals forces (S1 Table) but also has a hydrophobic interaction with Leu861(B). In summary, compared with arbidol, **Ar-2** not only interacts with more amino acid residues of the S protein but also forms polar interactions (hydrogen bonds with Arg765(B) and Ser596(A)), which enhances the binding ability of **Ar-2** to the S protein.

After the Br atom of **Ar-0** is replaced with a 4-sulfonamidophenyl group, **Ar-3** is obtained (Fig 13C). The 4-sulfonamidophenyl group also extends into hydrogen bonds and hydrophobic regions. On the one hand, it interacts with Leu303(A) and Tyr313(A) via Van Der Waals (S1 Table) forces; on the other hand, the sulfonamide group forms a strong hydrogen bond network with the guanidine group of Arg765(B) and the hydroxyl group of Thr302(A). In addition to the hydrophobic interactions between the methyl group of the benzene ring on the indolyl group and Val772(B), the interactions between the indole ring and its replaced groups of **Ar-3** and the surrounding amino acid residues are similar to those of arbidol. The introduction of the 4-sulfonamidophenyl group also causes a conformational change in the phenylthio group. The S atom of the phenylthio group has a hydrogen bond interaction with the hydroxyl group of Ser596(A), and the benzene ring has three types of interactions with its surrounding amino acid residues: (1) Van Der Waals (S1 Table) interactions with Leu611(A), Gln613(A), Ile666(A) and Lys733(B), (2) pi-alkyl hydrophobic interactions with Ala647(A) and Pro862(B), and (3) a pi-sigma interaction with Leu861(B). In summary, the interactions of the 4-sulfonamidophenyl and phenylthio groups with S1(A) and S2(B) greatly enhance the binding affinity of **Ar-3** with the S protein, and these increased interactions are significantly reflected in the high binding energy between **Ar-3** and the S protein.

## 4. Conclusion

In this study, AutoDock4.2, a molecular docking program, was used to perform molecular simulations with 14 drugs and the S protein, and these 14 drugs were demonstrated to have specific anti-SARS-CoV-2 effects. The results showed that tizoxanide, dolutegravir, bictegravir, and arbidol, each have a conformation capable of binding to key sites on the S protein with very high binding energies. Each tizoxanide molecule simultaneously interacts with the three functional fragments of the S protein and can “lock” S1(A), S1(B) and S2(C) mainly through hydrogen bonds and Van Der Waals forces so that the RBD of the metastable conformation of S1 before fusion cannot bind to ACE2; furthermore, each tizoxanide molecule interacts with the CH to affect fusion between S2 and host cells. In addition, tizoxanide forms a hydrogen bond with Cys379, which forms a disulfide bond and is in the core of S1, and the hydrogen bond may affect the stability of the S1 RBD. Bictegravir and dolutegravir, which are similar in structure, bind between the RBD and NTD of two adjacent S1 monomers and can limit the interaction between the RBD and ACE2 during the invasion of SARS-CoV-2 through various interactions. Arbidol binds to S1(A) and S2(B) mainly through nonpolar interactions, and these interactions can bind S1(A) and S2(B) tightly together. As a result, S1 cannot be shed when SARS-CoV-2 invades host cells, and the fusion function of S2 is also affected, rendering SARS-CoV-2 unable to invade host cells. Structure-based molecular optimization was further performed for the above four drugs. Molecular simulation of the optimized molecules reveals that nine molecules (three groups) can bind more effectively to their respective sites and that they not only have an increased binding energy but also can interact with more amino acid residues in different ways. Notably, all the atoms with relatively high polarity (O and N atoms) in **Ti-2** can form a strong hydrogen bond network with the surrounding amino acid residues through hydrogen bonds, **Ti-2** can simultaneously interact with S1(A), S1(B) and S2(C), and these interactions greatly enhance its binding energy with the S protein. **BD-2** not only binds to the RBD and NTD of two adjacent S1 monomers of the S protein but also interacts with HR1 of the S2 monomer with the NTD through Van Der Waals forces, indicating that **BD-2** can not only inhibit the binding between the RBD and ACE2 but also can affect the fusion between the S protein and host cells, which greatly enhances its ability to disrupt SARS-CoV-2. The sulfonamide group of **Ar-3** forms hydrogen bond interactions with Arg765(B) and Thr302(A), and the S atom also forms a hydrogen bond with Ser596(A); these new hydrogen bond networks are not observed in arbidol, which is one important reason for the increase in binding energy between **Ar-3** and the S protein. Overall, based on the above molecular simulations, molecular optimization, and interaction analysis between the molecules and the S protein, we conclude that tizoxanide, bictegravir, dolutegravir, and arbidol could effectively bind with the S protein. After further molecular optimization, the binding ability of the nine obtained new molecules (three groups) to the S protein was stronger than that of the original four molecules; therefore, they have better potential to inhibit the SARS-CoV-2 S protein. This study provides useful clues and inspiration for future studies on the discovery, optimization, and antiviral mechanisms of anti-SARS-CoV-2 drugs.

## Supporting information

S1 Table Van Der Waals forces formed between tizoxanide, dolutegravir, bictegravir, arbidol and their optimized products with the S protein. (DOC)

S1 Graphical abstract (TIF)

S1 File (DOCX)
