# Supplementary material for: Screening, simulation, and optimization design of small molecule inhibitors of the SARS-CoV-2 spike glycoprotein
Source: PLoS One. 2021 Jan 25;16(1):e0245975. doi: 10.1371/journal.pone.0245975 (PMC7833228; doi:10.1371/journal.pone.0245975)
Supplement: S1 Table — (DOC) [file pone.0245975.s001.doc]

**S1 Table.** Van Der Waals forces formed between tizoxanide, dolutegravir, bictegravir, arbidol and their optimized products with the S protein.

| Drugs and their optimized products | Van Der Waals energy (kcal/mol) |
| --- | --- |
| tizoxanide | -1.83 |
| dolutegravir | -2.63 |
| bictegravir | -2.72 |
| arbidol | -2.29 |
| Ti-1 | -2.77 |
| Ti-2 | -3.69 |
| Ti-3 | -3.53 |
| BD-1 | -4.67 |
| BD-2 | -4.27 |
| BD-3 | -4.13 |
| Ar-1 | -3.91 |
| Ar-2 | -3.04 |
| Ar-3 | -3.26 |
