# Supplementary figures and images for: Screening, simulation, and optimization design of small molecule inhibitors of the SARS-CoV-2 spike glycoprotein
Source: PLoS One. 2021 Jan 25;16(1):e0245975. doi: 10.1371/journal.pone.0245975 (PMC7833228; doi:10.1371/journal.pone.0245975)

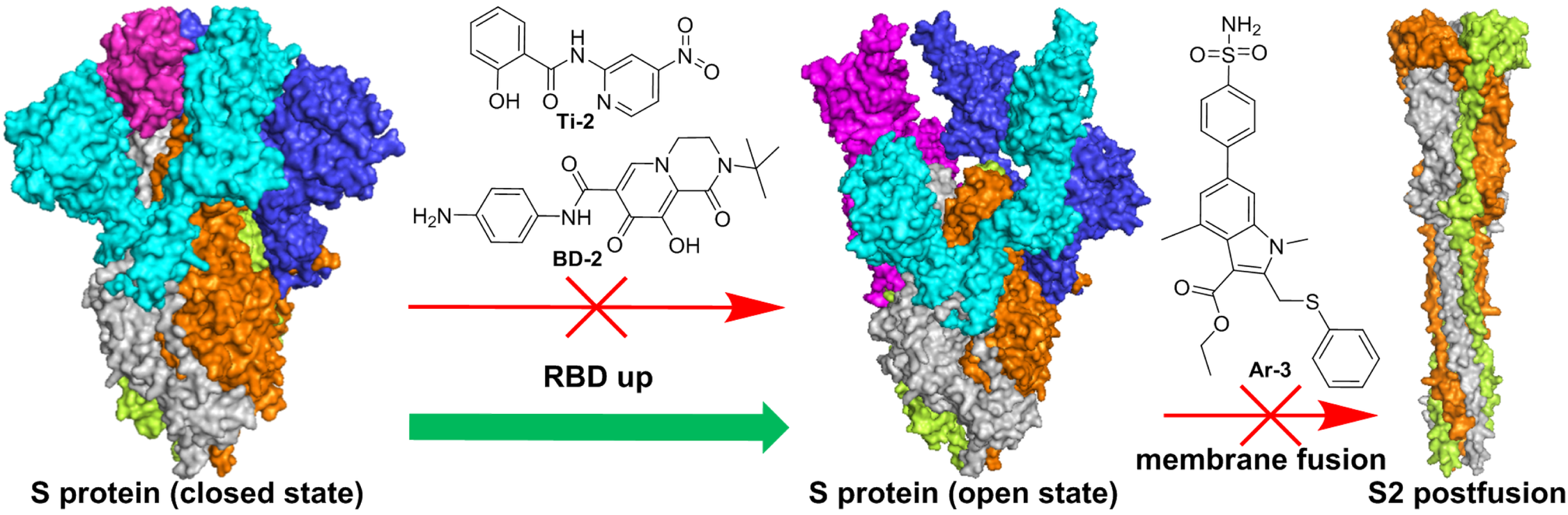

Supplement: S1 Graphical abstract — (TIF) [file pone.0245975.s002.tif]
