# Supplementary material for: Screening, simulation, and optimization design of small molecule inhibitors of the SARS-CoV-2 spike glycoprotein
Source: PLoS One. 2021 Jan 25;16(1):e0245975. doi: 10.1371/journal.pone.0245975 (PMC7833228; doi:10.1371/journal.pone.0245975)
Supplement: S1 File — (DOCX) [file pone.0245975.s003.docx]

- **Spike glycoprotein (S protein) of SARS-CoV-2 is a key target of antiviral drugs.**
- **Binding energy and interactions were calculated between 14 drugs and the S protein.**
- **Tizoxanide, dolutegravir, bictegravir, and arbidol effectively bound with S protein.**
- **These drugs may inhibit conformational changes between S1 and S2 of the S protein.**
- **Optimized Ti-2, BD-2, and Ar-3 have much stronger binding ability to the S protein.**
